# Response to ‘Chest compressions at altitude are of decreased quality, require more effort and cannot reliably be self-evaluated’

**DOI:** 10.1186/s13049-023-01158-x

**Published:** 2023-12-12

**Authors:** Maximilian Niederer, Dominik Roth, Alexander Egger

**Affiliations:** 1 Department of Anaesthesiology and Intensive Care Medicine, Hospital Scheibbs, Eisenwurzenstraße 26, Scheibbs, 3270 Austria; 2 Mountain Rescue Service Austria, Baumgasse 129, Wien, 1030 Austria; 3 Department of Emergency Medicine, Medical University of Vienna, Spitalgasse 23, Wien, 1090 Austria

To the editor,

We thank van Veelen and colleagues for their interest in our article on an ascent to high altitude on physical exhaustion during cardiopulmonary resuscitation (CPR) [1].

We wholeheartedly concur with the assessment that performing CPR under such unique circumstances requires greater effort and impairs providers’ ability to adhere to resuscitation guidelines at high altitudes. Whether the ascent was simulated through the use of a hypobaric chamber [2], made by car [3], or through an arduous ascent exceeding 1,200 m as in our case [1], the analysis of vital parameters showed pronounced exhaustion due to the demands of chest compressions at high altitude.

From this perspective, the findings by van Veelen et al. on providers’ struggle to reliably self-evaluate the quality of chest compressions at high altitudes is both interesting and significant. This is in line with our previous findings on the discrepancy between subjective exhaustion and actual quality of CPR at high altitude [4]. We could demonstrate that during ventilation phases, heart rate immediately decreases, even after 14 min of CPR, underlining the importance of frequent resting phases.

We also concur with the assessment of a need for widespread adoption of mechanical chest compression devices in alpine settings, as those have been shown to be viable, even in difficult terrain [5].

We fully endorse the call to adjust the guidelines for CPR in the alpine setting in the light of recent findings. There is a critical need to emphasize the widespread use of mechanical chest compression devices. In their absence, a minute-by-minute rotation of chest compressions might be advocated and should be further studied.

## References

[1] Niederer M, Tscherny K, Burger J (2023). Influence of high altitude after a prior ascent on physical exhaustion during cardiopulmonary resuscitation: a randomised crossover alpine field experiment. Scand J Trauma Resusc Emerg Med 24 Oktober.

[2] Vögele A, Van Veelen MJ, Dal Cappello Tet al. Effect of Acute Exposure to Altitude on the Quality of Chest Compression-Only Cardiopulmonary Resuscitation in Helicopter Emergency Medical Services Personnel: A Randomized, Controlled, Single‐Blind Crossover Trial. J Am Heart Assoc. 7. Dezember 2021;10(23):e021090. 10.1161/JAHA.121.021090 PMC9075389 34854317

[3] Narahara H, Kimura M, Suto T (2012). Effects of Cardiopulmonary Resuscitation at High Altitudes on the Physical Condition of untrained and unacclimatized rescuers. Wilderness Environ Med Juni.

[4] Egger A, Niederer M, Tscherny K (2020). Influence of physical strain at high altitude on the quality of cardiopulmonary resuscitation. Scand J Trauma Resusc Emerg Med Dezember.

[5] Egger A, Tscherny K, Fuhrmann V (2021). Comparison of different mechanical chest compression devices in the alpine rescue setting: a randomized triple crossover experiment. Scand J Trauma Resusc Emerg Med Dezember.

